# RNA-Seq Reveals miRNA Role Shifts in Seven Stages of Skeletal Muscles in Goat Fetuses and Kids

**DOI:** 10.3389/fgene.2020.00684

**Published:** 2020-07-07

**Authors:** Yinghui Ling, Qi Zheng, Jing Jing, Menghua Sui, Lu Zhu, Yunsheng Li, Yunhai Zhang, Ya Liu, Fugui Fang, Xiaorong Zhang

**Affiliations:** ^1^College of Animal Science and Technology, Anhui Agricultural University, Hefei, China; ^2^School of Natural and Environmental Sciences, Newcastle University, Newcastle upon Tyne, United Kingdom; ^3^Local Animal Genetic Resources Conservation and Biobreeding Laboratory of Anhui Province, Hefei, China

**Keywords:** goat (*Capra hircus*), miRNA, skeletal muscle, STEM, miRNA–mRNA network

## Abstract

MicroRNAs (miRNAs) are indispensable for the regulation of skeletal muscle. We performed RNA sequencing (RNA-seq) to establish a comprehensive miRNA profiling of goats in seven stages, namely, 45- (F45), 65- (F65), 90- (F90), 120- (F120), and 135-day (F135) fetuses, newborn (B1), and 90-day-old (B90) kids. In total, 421 known miRNAs and 228 goat novel miRNAs were identified in the data, and the average abundance of 19 miRNAs in seven stages exceeds 10,000 reads per million. Furthermore, 420 differentially expressed miRNAs (DEmiRNAs) were identified in all comparison group at seven stages, 80 of which were uniquely differentially expressed in the B1 and B90 comparison groups. Pathway analysis indicated that this group was associated with the release of muscle hypertrophy and regulation of myoblast proliferation. Besides, 305 DEmiRNAs were clustered into three significantly enriched profiles (profiles 11, 16, and 19). Function analysis revealed that profile 16 was related to muscle hypertrophy and differentiation. Profile 11 was involved in multiple enzyme activities and metabolic processes in muscle cells. And profile 19 was involved in material transport and structural stability. Two highly expressed miRNAs and three key miRNAs (chi-miR-328-3p, chi-miR-767, and chi-miR-150) of these profiles were verified to be consistent with the data by quantitative real-time PCR. These results provided a catalog of goat muscle-associated miRNAs, allowing us to better understand the transformation of miRNA roles during mammalian muscle development.

## Introduction

Skeletal muscle is a complex and important tissue that occupies 40% of mammals’ body weight (Jung et al., 2019). The development of embryonic skeletal muscle tissue is mainly manifested by an increase in the number of muscle fibers. The myoblasts from the mesoderm first migrate to the limbs and trunk and rapidly proliferate (Endo, 2015; Chal and Pourquie, 2017). After reaching a certain number, myoblasts differentiate into myocytes and fuse into multinucleated myotubes (Endo, 2015; Ganassi et al., 2018). The fetal period is the main period of muscle formation, and the number of muscle fibers remains constant after birth (Zhu et al., 2004; Wang et al., 2010). After the mammal is born, the growth of skeletal muscle tissue mainly depends on the circumference and length of the muscle fiber, also known as muscle fiber hypertrophy (Al-Shanti and Stewart, 2009; Murach et al., 2018). Therefore, there are significant differences in the development of skeletal muscles before and after birth. And the transformation of skeletal muscle growth and development is essentially the result of gene regulation. MicroRNAs (miRNAs), the non-coding single-stranded RNA of 19–25 nucleotides, are emerging as robust players of gene regulation (Adlakha and Saini, 2014). Most of the miRNAs in animals mainly bind to the 3’-UTR, 5’-UTR, or CDS sequence of their target mRNA through their 5’-end two- to eight-seed sequences, resulting in interference or degradation of the target mRNA (Meister, 2007; Helwak et al., 2013). Thus, they are involved in various biological processes, such as cell proliferation (Roy et al., 2017), apoptosis (Gorbea et al., 2017), differentiation (Title et al., 2018), metabolism (Antoniali et al., 2017), and tumor metastasis (Fang et al., 2018). And miRNAs have different expression patterns before and after animal birth in skeletal muscle (Zhao et al., 2016). Analysis of this difference will help in the further study of the regulation mechanism of miRNAs on animal skeletal muscle development.

Goat, one of the most basic commercial animals, has attracted increasing attention as a viable source of meat. However, goat has lower meat production than other commercial animals, which hinders the development of goat industries. Some studies have shown that miRNAs have an indispensable role in goat muscle development at different periods. For example, miR-27b was identified to inhibit the proliferation but promoted differentiation of skeletal muscle satellite via targeting PAX3 (Ling et al., 2018). And miR-487b-3p, which is significantly higher in the fetus than in adult, suppressed myoblast proliferation and differentiation by targeting IRS1 in skeletal muscle myogenesis (Wang et al., 2018).

In our study, seven developmental stages of Anhui white goat skeletal muscle were used for RNA sequencing (RNA-seq) analysis, namely, 45- (F45), 65- (F65), 90- (F90), 120 (F120), and 135-day (F135) fetuses and kids born within 24 h and 90 days (B1 and B90). miRNAs (known miRNAs and goat novel miRNAs) were systematically identified from multiple types of small RNAs (sRNA), and functions and pathways of dynamic expression profiles were elucidated. These findings revealed the molecular roles of miRNAs and the mechanisms operating at different stages of mammalian skeletal muscle development.

## Materials and Methods

### Sample Collection

A group of female Anhui white goats managed and raised in the Hefei Boda Animal Husbandry Technology Development Co., Ltd (Hefei, Anhui, China), was selected and treated with EAZI-Breed CIDR (CIDR, Hamilton, New Zealand) for 12 days for simultaneous estrus. The female goats with successful estrus were selected for artificial insemination. Fertilization was observed regularly. Twenty-one pregnant goats were used to obtain samples, each stage with three goats. Before sample collection, all the experimental animals (including female goats, fetuses, and kids) were injected with Jingsongling (2,4-xylyl xylazole, lot number 030725, produced by Shandong Zibo Veterinary Medicine Factory, Shandong, China) in the hips at a dose of 2 mg kg^–1^ for deep anesthesia. Under complete anesthesia, all experimental samples were killed by arterial bleeding with the help of slaughterhouse professionals (this method complied with the euthanasia guide of the Chinese Society of Laboratory Animals, no. T/CALAS 31-2017). Fetuses were obtained from goats that had been pregnant for 45, 65, 90, 120, and 135 days by cesarean section. Kids that are 1- and 90-day-old were also collected. The *longissimus dorsi* muscles of the seven stages of goat development were collected as experimental samples. Each stage had three biological replicates. After collection, the samples were washed three times with PBS and placed in liquid nitrogen to be stored frozen.

### Library Preparation and Sequencing of Skeletal Muscle

Total RNA isolation was performed using TRIzol reagent (Invitrogen, Carlsbad, CA, United States) on 21 *longissimus dorsi* muscle samples. Then, RNA concentration was measured using the Qubit^®^ RNA Assay Kit in Qubit^®^ 2.0 Fluorometer (Life Technologies, Carlsbad, CA, United States), RNA degradation and contamination were checked with 1% agarose gels, and RNA integrity was assessed using the RNA Nano 6000 Assay Kit (Agilent Technologies, Santa Clara, CA, United States) of the Agilent Bioanalyzer 2100 system (Agilent Technologies, Santa Clara, CA, United States). A total amount of 3 μg total RNA per sample was used as input material for the sRNA sequencing libraries generated using NEBNext^®^ Multiplex Small RNA Library Prep Set for Illumina^®^ (NEB, Ipswich, MA, United States), and index codes were added to attribute sequences to each sample, followed by reverse transcription. PCR amplification was performed using LongAmp Taq 2X Master Mix, SR Primer for Illumina, and index (X) primer. PCR products were purified on an 8% polyacrylamide gel (100 V, 80 min). The small non-coding RNA plus the 3’ and 5’ adaptors were 140–160 bp in length, and the gel of these fractions was recovered and dissolved in 8 μl of elution buffer. Then, the quality of libraries was calculated by a DNA high-sensitivity chip on the Agilent Bioanalyzer 2100 system. Finally, the clustering of the index-coded samples was performed on a cBot Cluster Generation System using a TruSeq SR Cluster Kit v3-cBot-HS (Illumina), and the library preparations were sequenced on an Illumina HiSeq 2500 platform, and 50-bp single-end reads were generated.

### Data Calculation of Small RNA

Raw data in a fastq format were first processed through custom Perl and Python scripts. Raw data filtered reads contain ploy-N (with 5’ adapter contaminants and ploy A or T or G or C, without 3’ adapter or the insert tag) and low-quality reads to obtain clean data. The mismatched miRNA tags were mapped to the reference sequence^1^ by Bowtie (Langmead et al., 2009). Then, the 18- to 35-nt fragments were selected from clean data for subsequent analyses. And the expression and distribution were analyzed according to the reference. Some sRNA reads map to multiple categories. In order to map each sRNA to only one annotation, we set the following precedence rule: known miRNA > rRNA > tRNA > snRNA > snoRNA > repeat > gene > novel miRNA.

### Known and Goat Novel miRNA Alignments and Annotations

Small RNA reads were mapped to obtain known miRNAs. The total rRNA proportion was used as a marker as sample quality indicator. The acquisition of potential miRNAs and the mapping of secondary structures were performed by the modification software miRDeep2 (Friedlander et al., 2012) with miRBase 21.0 as a reference. sRNA tags were mapped to RepeatMasker, and the Rfam database was used to remove tags originating from protein-coding genes (intro ± link and exon ± link), repeat sequences, rRNA, tRNA, snRNA, and snoRNA. Moreover, the available software miREvo (Wen et al., 2012) and miRDeep2 (Friedlander et al., 2012) explored the secondary structure of unannotated fragments. And the Dicer cleavage site also has the minimum free energy of sRNA tags to predict goat novel miRNAs in the previous steps.

### Analysis of miRNA

miRNA families were identified from samples in other species. In our analysis pipeline, goat novel miRNA precursors were submitted to Rfam^2^ to look for Rfam families.

Reads per million (RPM) was used as a unit for estimating the level of miRNA (Zhou et al., 2010). Differential expression analysis of two stages was performed by the DESeq R package (1.8.3). The *P*-value was adjusted using the Benjamini-and-Hochberg method. A corrected *P*-value (*P*-adj) < 0.05 of miRNA was considered a differential expression. Bubble charts were constructed using the OmicShare platform for data analysis^3^. And heat map analysis was formed using Morpheus^4^.

### Functional and Pathway Analysis of miRNA Targets

The target genes of miRNAs were predicted using miRanda. Function and pathway differentially expressed miRNA (DEmiRNAs) target genes were demonstrated using Gene Ontology (GO) and the Kyoto Encyclopedia of Genes and Genomes (KEGG). GOseq-based Wallenius non-central hypergeometric distribution, which could be adjusted for gene length bias, was implemented for GO analysis (Young et al., 2010). KOBAS software was used to calculate candidate enrichment of target genes in the KEGG pathway (Mao et al., 2005).

### Quantitative Real-Time PCR

Reverse transcription of selected DEmiRNAs was performed using a GoScript Reverse Transcriptase kit (Promega, Madison, WI, United States) according to the manufacturer’s guide. All of the primer pairs (Table 1) were designed using NCBI and synthesized by the Shanghai General Biotech Co. Ltd (Table 1). *U6* was amplified as a control. Next, GoTaq qPCR Master Mix (Promega, catalog number: A6002, Madison, WI, United States) was used to perform quantitative real-time PCR (qRT-PCR) on a LightCycler 96 (ABI) real-time PCR instrument according to the manufacturer’s instructions. The PCR mixture included 7.5 μl SYBR Real-Time PCR System, 1.5 μl cDNA, and 1 μl forward and reverse primers (total 10 μM). The reaction was denatured at 95°C for 2 min, followed by 40 cycles of 95°C for 15 s, 60°C for 1 min, and an extension at 60°C for 30 s, and then recovered at a rate of 0.2°C per 1 s to 95°C; finally, it was stored at room temperature for 10 s. Each sample was run in triplicate. The target sequence quantity was normalized to the reference sequence and calculated as 2^–ΔΔCt^. The Windows version of SPSS 21.0^5^ was used for statistical analysis of standardized data. Data were presented as means ± SE and were considered statistically significant if the *P-*value was <0.05.

**TABLE 1:** Sequences of the primers used for qRT-PCR analysis.

Gene name	Primer sequence
chi-miR-1	Reverse (R): GTCGTATccagtgcagggtccgaggtaTTCGCACT GGATACGACATACAT
	Forward (F): CACGCATGGAATGTAAAG
chi-miR-381	R: GTCGTATccagtgcagggtccgaggtaTTCGCACTGGAT ACGACACAGAG
	F: CACGCATATACAAGGGCA
chi-miR-328-3p	R: GTCGTATccagtgcagggtccgaggtaTTCGCACTGGAT ACGACACGGAA
	F: CACGCACTGGCCCTCTCT
chi-miR-767	R: GTCGTATccagtgcagggtccgaggtaTTCGCACTGGATA CGACATGCTC
	F: CACGCATGCACCATGGTT
chi-miR-150	R: GTCGTATccagtgcagggtccgaggtaTTCGCACTGGATA CGACCACTGG
	F: CACGCATCTCCCAACCCT
*U6*	R: CTCAGAATCACCCAATGC
	F: ATGTTCATCCAGTTGTCAC

## Results

### Overview of Seven-Stage Skeletal Muscle miRNA Sequencing Data

**FIGURE 1:**
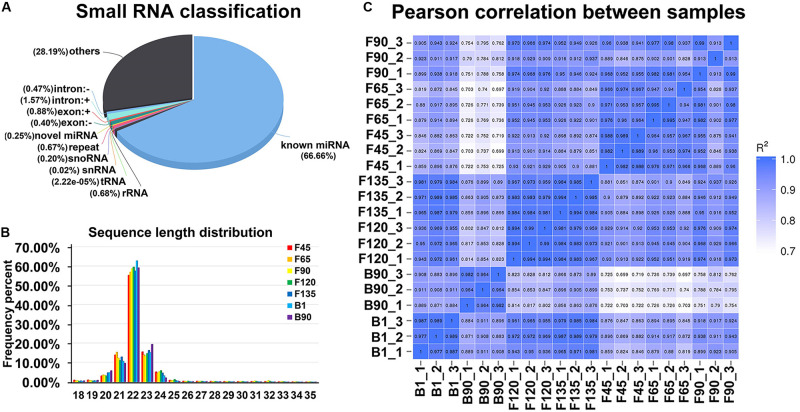
Global small RNA analysis of goat skeletal muscle in seven developmental stages. **(A)** The classification of small RNAs for goat skeletal muscle. Small RNA classification was performed using the priority of “known miRNA > rRNA > tRNA > snRNA > snoRNA > repeat > gene > novel miRNA.” Others represent samples aligned to the reference sequence, but no alignment to the small RNA shown in the figure. **(B)** Percentage distribution of all small RNA sequence lengths. Characteristics of known and goat novel miRNAs in goat skeletal muscle. **(C)** Heat map of replicate samples of the same stage of goat skeletal muscle. The color spectrum, ranging from white to blue, represents Pearson correlation coefficients ranging from 1 to 0.7, indicating high to low correlations.

To identify miRNAs involved in early skeletal muscle development, sRNA libraries were constructed from total RNA of 21 individuals in seven stages. The libraries generated in seven developmental stages ranged from 10,217,979 to 15,858,768 raw reads, with an average of 13,409,245 reads per stage. After discarding adaptors and low-quality reads, an average of 13,180,785 clean reads was obtained per sample of the 21 sRNA libraries (Supplementary Table S1). After length screening, a total of 12,910,674 sRNA reads were obtained from clean reads. Furthermore, 84.6–96.26% of the sRNA could be mapped to the reference sequence in the sRNA libraries (Supplementary Table S1). Subsequently, the annotated sRNAs varying from 18 to 35 nt were divided into several categories: known miRNA (66.6%), intro (+ and - link, 2.04%), exon (+ and - link, 1.28%), rRNA (0.68%), snoRNA (0.20%), novel miRNA (0.25%), snRNA (0.02%), and others (28.19%) (Figure 1A). Among them, known miRNAs accounted for the largest proportion. In addition, the obtained sRNAs were counted in length. Their lengths were concentrated at 20–24 nt (Figure 1B). These data validated the reliability of the experiment. And there was a high correlation within samples at each stage, which also verified the reliability between samples at the same stage (Figure 1C).

**FIGURE 2:**
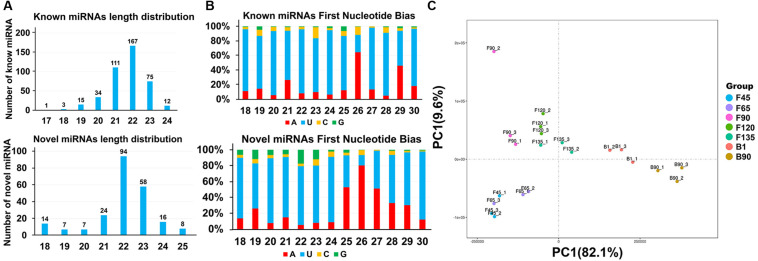
Characterization of all identified miRNAs. **(A)** Length distribution of known miRNAs **(top)** and goat novel miRNAs **(below)**. **(B)** Percentage of base preference distribution for known miRNAs **(top)** and goat novel miRNAs **(below)**. **(C)** PCA of seven stages of goat skeletal muscle miRNAs.

A total of 421 known miRNAs and 228 goat novel miRNAs were obtained in the data, and their lengths were concentrated between 21 and 23 nt (Supplementary Table S2 and Figure 2A). Due to the specificity of the cleavage site, the first base of the miRNA mature sequence had a strong bias. And the main base preferences were U and A, which also verified the reliability of the data (Figure 2B). Principal component analysis (PCA) of all detected miRNA showed that the developmental order could be accurately captured from F45 to B90, and there was a segmentation before and after birth (Figure 2C). Pearson correlation analysis of all 21 sample pairs demonstrated similar results (Figure 1C). These two calculations showed the same cluster expression profiles for the identified miRNAs.

### Transcriptional Dynamics of Differentially Expressed miRNA

**FIGURE 3:**
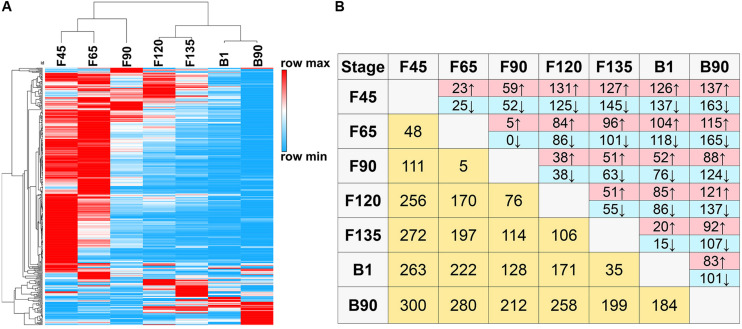
DEmiRNAs in seven goat skeletal muscle developmental stages. **(A)** Hierarchical clustering heat map of all DEmiRNAs by samples. **(B)** Number of DEmiRNAs showing upregulation (red) or downregulation (blue) during development. Light yellow: number of total DEmiRNAs between two stages.

To understand the functional transformation of miRNAs at different stages in the skeletal muscle, it is essential to study the expression profiles of DEmiRNAs in skeletal muscle development at different stages. A total of 420 DEmiRNAs (*P*-adj < 0.05, including 364 known and 56 novel DEmiRNAs) were obtained in comparison groups of any two phases (Supplementary Table S3). The unbiased hierarchical clustering of DEmiRNAs indicated that the largest segmentation occurred between F90 and F120 (Figure 3A). Moreover, in the comparison groups of two consecutive stages, F90 and F120 comparison groups also have the most DEmiRNAs (Figure 3B). There were 76 DEmiRNAs observed between F90 and F120, of which 38 were upregulated and 38 were downregulated (Figure 3B). Besides, the maximum number of DEmiRNAs (300) found between F45 and B90 and the minimum number of DEmiRNAs (5) were observed between F65 and F90, including chi-miR-136-5p, chi-miR-543-3p, chi-miR-497-5p, chi-miR-15b-3p, and chi-miR-34b-3p (Figure 3B).

### Stage-Specific DEmiRNA of Developmental Transition

**FIGURE 4:**
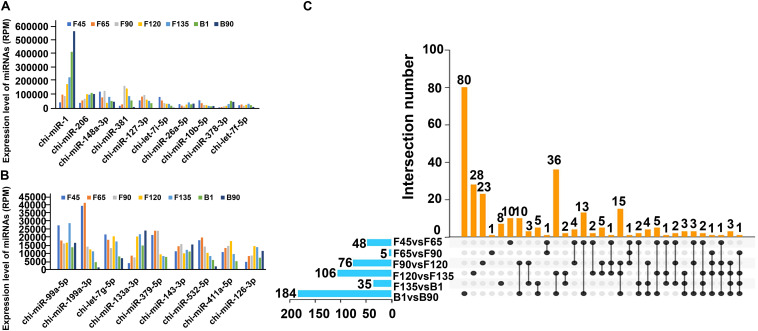
Stage-specific dynamics of miRNAs expression in seven stages of goat skeletal muscle. **(A,B)** DEmiRNA expression level display with RPM > 10,000. **(C)** UpSet Venn diagram of DEmiRNAs in the skeletal muscle. The horizontal histogram on the left shows the total number of DEmiRNAs in each comparison group. The matrix in the middle represents different sets. There is only one black dot in the same column, indicating the set expressed only in the group **(left)**. The same column has two black dots, indicating the intersection of these two groups. Three black dots in the same column represent the intersection of these three groups and so on. The histogram on the right is the number of DEmiRNAs in the corresponding set.

To determine the role of DEmiRNAs throughout the seven developmental stages, we identified DEmiRNAs that were highly expressed at all stages. The total expression level of DEmiRNAs in seven stages varied between 0.14 and 1,632,833.52 with an intermediate value of 171.26 (Supplementary Table S3). Among them, 33.73% (142) DEmiRNAs showed high-abundance expression in seven stages (mean total RPM > 1,000), and the average expression level of 19 miRNAs in seven stages exceed 10,000 RPM, including chi-miR-1, chi-miR-206, chi-miR-148a-3p, chi-miR-381, chi-miR-127-3p, chi-let-7i-5p, chi-miR-26a-5p, chi-miR-10b-5p, chi-miR-378-3p, chi-let-7f-5p, chi-miR-99a-5p, chi-miR-199a-3p, chi-let-7g-5p, chi-miR-133a-3p, chi-miR-379-5p, chi-miR-143-3p, chi-miR-532-5p, chi-miR-411a-5p, and chi-miR-126-3p (Supplementary Table S3 and Figures 4A,B). And miR-1 had the highest expression in all stages, accounting for 23.36% of all expressions (Supplementary Table S3). Their constant expression suggested that they may perform essential cellular biological functions during skeletal muscle development to maintain cell integrity. Besides, we analyzed DEmiRNAs that were uniquely expressed in successive developmental stages to determine the unique role of DEmiRNAs at the seven stages (Figure 4C). Specifically, 80 DEmiRNAs were found only in B1 and B90, precluding the prenatal DEmiRNAs (Supplementary Table S4 and Figure 4C). Among these DEmiRNAs, 34 and 46 were upregulated and downregulated, respectively. The upregulated DEmiRNAs were mainly involved in “HIF-1 signaling pathway,” “ErbB signaling pathway,” and other pathways that are associated with skeletal muscle development. Conversely, downregulated DEmiRNAs were involved in the “regulation of actin cytoskeleton,” “fatty acid biosynthesis,” and others pathways, which were related to intramuscular fat development (Supplementary Table S5). The earliest comparison group (F45 vs F65) participated in the “glycerophospholipid metabolism,” “GnRH signaling pathway,” and “citrate cycle (TCA cycle)” (Supplementary Table S5). Likewise, 23 DEmiRNAs were only found in the F90 and F120 comparison groups and were involved in “ribosome,” “Ras signaling pathway,” “rap1 signaling pathway,” and “glutathione metabolism” (Supplementary Tables S4, S5 and Figure 4C). And 28 DEmiRNAs only expressed between F120 and F135, which were involved in “SNARE interactions in vesicular transport,” “hedgehog signaling pathway,” “glycosaminoglycan biosynthesis—heparan sulfate/heparin,” and “lysosome” (Supplementary Tables S4, S5 and Figure 4C). In addition, eight DEmiRNAs in the F135-vs-B1 comparison group participated in the most signaling pathways, among which are “glycine, serine and threonine metabolism,” “arginine and proline metabolism” associated with amino acid metabolism, and “fatty acid degradation,” “fat digestion and absorption,” and “glycerolipid metabolism” associated with intermuscular fat production (Supplementary Tables S4, S5 and Figure 4C). These DEmiRNAs may represent the initiation/termination of physiological processes at a particular developmental stage in the skeletal muscle.

### Trend Analysis Reflects the Role of miRNAs in Goat Skeletal Muscle

**FIGURE 5:**
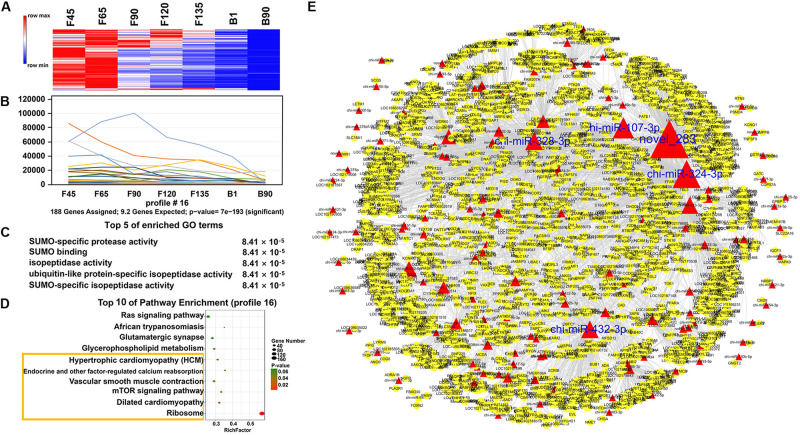
Expression pattern, functional analysis, and miRNA–mRNA network of profile 16. **(A)** Heat map of profile 16 miRNAs across all seven stages. **(B)** Expression trend of profile 16. **(C)** The top five GO terms of profile 16 and corresponding enrichment *P-*values are shown on the right side. **(D)** The top 10 KEGG enrichment terms of profile 16. The bubble size represents the number of genes contained. *P*-value is from 0 to 0.06; red represents low *P*-value, and green represents high *P*-value. The yellow box represents the pathway with a *P*-value < 0.05. **(E)** miRNA–mRNA network for profile 16. The red triangles represent the DEmiRNAs, the yellow circles represent mRNAs, and the size represents the number of miRNA target genes. The larger the size, the greater the number of miRNA target genes. The blue word represents the top five nodes and is also the key miRNAs.

**FIGURE 6:**
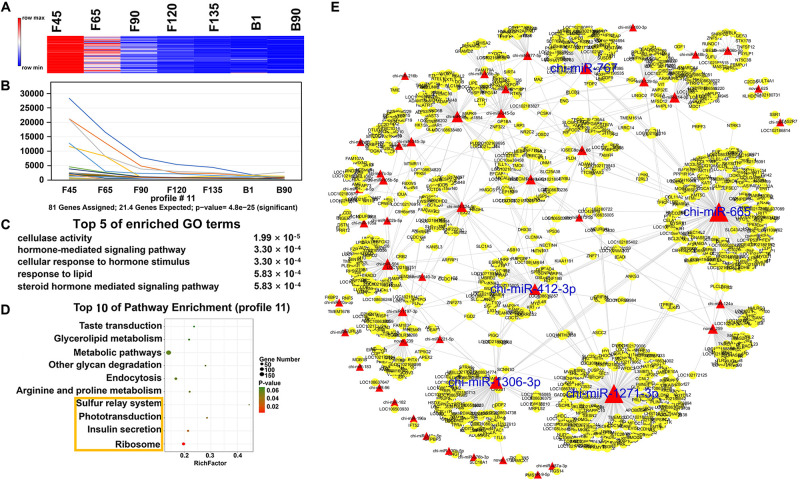
Expression pattern, functional analysis, and miRNA–mRNA network of profile 11. **(A)** Heat map of profile 11 miRNAs across all seven stages. **(B)** Expression trend of profile 11. **(C)** The top five GO terms of profile 11 and corresponding enrichment *P-*values are shown on the right side. **(D)** The top 10 KEGG enrichment terms of profile 11. The bubble size represents the number of genes contained. *P*-value is from 0 to 0.06; red represents low *P*-value, and green represents high *P*-value. The yellow box represents the pathway with a *P*-value < 0.05. **(E)** miRNA–mRNA network for profile 11. The interpretation of the miRNA–mRNA network is the same as in Figure 5.

**FIGURE 7:**
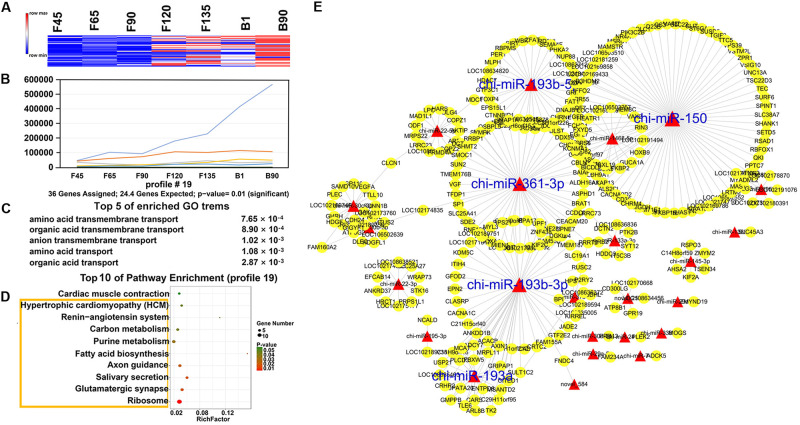
Expression pattern, functional analysis, and miRNA–mRNA network of profile 19. **(A)** Heat map of profile 19 miRNAs across all seven stages. **(B)** Expression trend of profile 19. **(C)** The top five GO terms of profile 19 and corresponding enrichment *P-*values are shown on the right side. **(D)** The top 10 KEGG enrichment terms of profile 19. The bubble size represents the number of genes contained. *P*-value is from 0 to 0.05; red represents low *P*-value, and green represents high *P*-value. The yellow box represents the pathway with a *P*-value < 0.05. **(E)** miRNA–mRNA network for profile 19. The interpretation of the miRNA–mRNA network is the same as in Figure 5.

In order to understand the dynamic changes of DEmiRNA levels during goat skeletal muscle development more clearly, we selected DEmiRNAs with *P*-adj < 0.05 and |fold change| > 2 for trend analysis. A total of 305 DEmiRNAs were clustered into 12 expression patterns, three of which were significantly enriched, including profiles 16, 11, and 19 (Supplementary Figure S1). Profile 16 contained the most DEmiRNAs (188) and had a high expression level before birth (Figures 5A,B). They were enriched (*P* < 0.05) in 242 GO terms that mainly related to various enzymes, such as “SUMO-specific protease activity,” “SUMO binding,” and “isopeptidase activity” (Figure 5C and Supplementary Table S6). And they were involved in six enriched (*P* < 0.05) pathways, including “ribosome,” “mTOR signaling pathway,” “endocrine and other factor-regulated calcium reabsorption,” and other pathways (Figure 5D and Supplementary Table S7). Among the DEmiRNAs in profile 16, 126 DEmiRNAs targeted 2,903 mRNAs. Five DEmiRNAs with the most target genes were identified as key DEmiRNAs for this profile, namely, novel-283, chi-miR-107-3p, chi-miR-328-3p, chi-miR-324-3p, and chi-miR-432-3p (Figure 5E). Profile 11 (81 DEmiRNAs), which gradually decreased in all stages, targeted 1,340 mRNAs (Figures 6A,B). Functional analysis revealed that these target genes were enriched in 110 terms, 54 of which were related to response, enzyme activity, and the metabolic process, including “cellulase activity,” “cellular response to hormone stimulus,” and “cellular response to steroid hormone stimulus” (Figure 6C and Supplementary Table S8). Besides, some of the enriched functional terms of the skeletal muscle associated with proliferation, such as “cell proliferation,” “intermediate filament,” and “intermediate filament cytoskeleton” (Supplementary Table S8). And pathway analysis was enriched in “ribosome,” “insulin secretion,” “phototransduction,” and “sulfur relay system” (Figure 6D and Supplementary Table S9). The miRNA–mRNA network of profile 11 showed five key miRNAs, namely, chi-miR-665, chi-miR-412-3p, chi-miR-1271-3p, chi-miR-1306-3p, and chi-miR-767 (Figure 6E). Furthermore, profile 19 contained 36 DEmiRNAs with 301 targeting mRNAs, and their expression levels rose in all stages (Figures 7A,B). The 58 functional enriched terms are related to material transport and structural stability, including “amino acid transmembrane transport,” “maintenance of protein location,” and “cytoskeleton organization” (Figure 7C and Supplementary Table S10). The pathways were enriched in “ribosome,” “axon guidance,” “fatty acid biosynthesis,” and others (Figure 7D and Supplementary Table S11). The five key miRNAs of profile 19 were chi-miR-150, chi-miR-361-3p, chi-miR-193b-5p, chi-miR-193b-3p, and chi-miR-193a (Figure 7E).

### QRT-PCR Validation of DEmiRNAs

**FIGURE 8:**
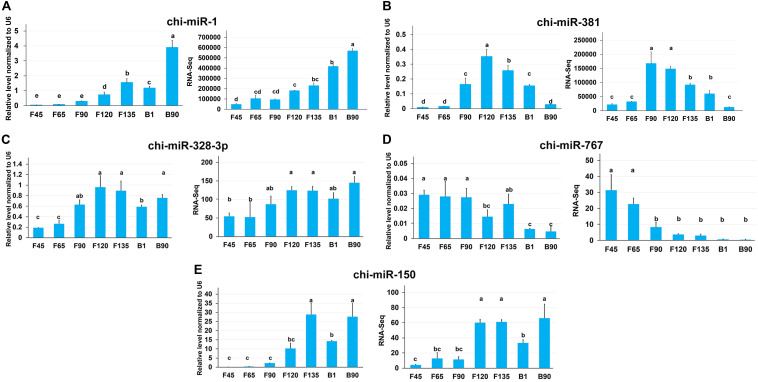
The expression of miRNAs in seven stages of skeletal muscle development was validated by qRT-PCR. **(A)** qRT-PCR expression level **(left)** and RNA-seq expression level **(right)** of chi-miR-1 and chi-miR-381 that highly expressed in all stages. **(B–D)** Key miRNAs were screened in each expression profile for qRT-PCR validation.

To validate data from RNA-seq, we validated five DEmiRNAs using qRT-PCR, including two miRNAs with high expression and one key miRNA in each expression profile. All of the verified miRNA expression levels showed a trend consistent with the amount of RNA-seq expression (Figure 8). miR-1 maintained a high expression level after F135, while miR-381 had a higher expression level from F120 to B1 (Figures 8A,B). In addition, chi-miR-328-3p, chi-miR-767, and chi-miR-150 were the key miRNAs of profiles 16, 11, and 19, respectively, and their qRT-PCR expression and RNA-seq expression were also consistent (Figures 8C–E).

## Discussion

As a branch of non-coding RNAs, miRNAs actively participate in the regulation of skeletal muscle development. With the development of sequencing technology, it is found that miRNAs in animal transcriptomes are more abundant than previously thought. There are now thousands of such molecules in skeletal muscles of many species, such as humans (Horak et al., 2016), sheep (Liu et al., 2019), and goat (Guo et al., 2016). Horak et al. (2016) reviewed several representative miRNAs found in basic research and sequencing that were specifically expressed in skeletal muscle and played an important role in proliferation, differentiation, and regeneration, including miR-1, miR-133a, miR-133b, miR-206, miR-208b, miR-486, and miR-499. These miRNAs are DEmiRNAs in this study. Liu et al. (2019) analyzed the GO and KEGG enrichment terms for the 345 DEmiRNAs of sheep’s *longissimus dorsi* between fetuses and adults and showed the regulatory network of 29 DEmiRNAs and 298 target genes. In addition, Wang et al. (2014) revealed the expression profiles of goat miRNAs in the fetal and 6-month-old stages, identified 336 DEmiRNAs, and found that miR-424-5p and miR-29a may regulate muscle development. Furthermore, Guo et al. (2016) explored the miRNA expression profiles and their characteristics in four stages of goat skeletal muscle and the functions and pathways of DEmiRNA (RPM > 100) target genes at each stage of transformation. In addition, the relative weight gain rate of goats was accelerated before 90 days, while the absolute weight gain was accelerated after 90 days, especially after 120 days (Zhao et al., 2012). And miRNAs may change in the skeletal muscle before and after birth. Therefore, we chose seven-stage AWGs, namely, F45, F65, F90, F120, F135, B1, and B90. In our study, we not only analyzed the expression profile of miRNAs during the seven skeletal muscle developmental stages before and after goat birth and explored the possible role of miRNA target genes in each transformation stages and clustered trend expression patterns of DEmiRNAs but also performed functional and pathway analyses of enrichment trends to better understand the possible role of miRNAs with the same trend throughout development and screened out key miRNAs in each trend based on the miRNA–mRNA network.

All identified miRNAs (421 known miRNAs and 228 goat novel miRNAs) were concentrated between 21 and 23 nt, and bases were at most U, which was consistent with reported characteristics of miRNAs (Bartel, 2004). From this, we verified the reliability of the data and then analyzed the differences. A total of 420 DEmiRNAs were found in any two comparison groups. Among them, 19 DEmiRNAs expressed more than 10,000 RPM. Many of these miRNAs were involved in muscle development. miRNA-1 and miRNA-133a served as examples; they mediated the Dlk1-Dio3 Mega gene cluster inhibition to achieve metabolic maturation during muscle stem cell differentiation (Wust et al., 2018). miRNA-1 and miRNA-133a-3p were expressed at a low level in the early stage and an extremely high level after F120, indicating that they were essential factors regulating muscle differentiation and maturation after F120. And miR-206 promoted myoblast differentiation and muscle fiber hypertrophy, and it played an active role in the treatment of skeletal muscle injury (Ma et al., 2015). miR-206 was also highly expressed after F120, indicating that the progresses of differentiation and hypertrophy of goat skeletal muscle increased at F120. In addition, miR-148a-3p regulated proliferation and apoptosis of bovine muscle cells by targeting KLF6 (Song et al., 2019). It had a high expression level in the early stage, indicating that it might promote muscle cell proliferation and inhibited the process of apoptosis before goat F120. These results suggested that miRNAs that highly expressed in F45 to B90 may have an essential effect on skeletal muscle development.

To determine the unique role of DEmiRNAs in the seven developmental stages of skeletal muscles, we analyzed the unique DEmiRNAs in two consecutive stages of comparison groups. The most diverse DEmiRNAs in the B1 and B90 comparison groups are upregulated in the “HIF-1 signaling pathway,” “ErbB signaling pathway,” and others. The ErbB signaling pathway was thought to play an important role in myocyte proliferation, and the HIF-1 signaling pathway improved cell hypertrophy (Lebrasseur et al., 2003; Tan et al., 2010; Shang et al., 2013). This suggested that upregulated miRNAs in the B1 and B90 comparison groups that were uniquely expressed after birth released hypertrophic inhibition and regulated myocyte proliferation. The “TCA cycle” of the earliest comparison group (F45 vs F65) helped maintain muscle energy requirements and tissue homeostasis (Capitanio et al., 2017). The Ras signaling pathway involved in F90 and F120 comparison groups also played an important role in skeletal muscle development. The dysregulated Ras/MAPK pathway activity directly inhibited skeletal muscle development (Tidyman et al., 2011). The F135-vs-B1 comparison group was involved in a variety of amino acid signaling pathways, including glycine, serine, and threonine metabolism, all of which played an important role in skeletal muscle cells (Ost et al., 2015; He et al., 2017; Chen et al., 2018). For example, serine/threonine kinase 40 (Stk40) acted as a positive regulator of skeletal myoblast differentiation in culture and fetal skeletal muscle formation (He et al., 2017).

Finally, we clustered all miRNAs and found three major trends, namely, profiles 11, 16, and 19. For profile 16, they have a high level of expression in the prenatal phase. The enrichment of GO and KEGG may mean that prenatal miRNAs have reduced inhibition of skeletal muscle hypertrophy and differentiation-related pathways, and the mRNA expression associated with these pathways begins to rise. chi-miR-107-3p had a high expression level (RPM > 100) before F120, which can target 173 mRNAs. For example, its target gene *DLL-1* interacts with the NOTCH receptor to reduce the transmission of Notch signaling, leading to skeletal muscle hypertrophy (Al Jaam et al., 2016). Another target gene, *DOCK3*, that overexpressed it could regulate PTEN/AKT signaling, thereby regulating muscle hypertrophy and growth and inducing apoptosis (Alexander et al., 2014). chi-miR-324-3p, another key miRNA of profile 16, targeted *AKT1*, which promoted type IIb muscle growth (Cheng et al., 2015). And *AKT1* also promoted load-induced muscle hypertrophy by regulating satellite cell proliferation (Moriya and Miyazaki, 2018). Besides, inhibition of chi-miR-328-3p targeting gene *PLD* prevented mechanically induced PA elevation and activation of mTOR signaling (Hornberger et al., 2006). Moreover, novel-283 targeted *AMOT* and *BCL9*. Amot was a necessary factor to regulate the expansion and self-renewal of muscle stem cells (Li and Fan, 2017). BCL9, as an essential component of canonical Wnt signaling, mediated the differentiation of myogenic progenitors during muscle regeneration (Brack et al., 2009). And chi-miR-432-3p targeted NPRL2, and its reduced expression level would make muscle fibers larger (Dutchak et al., 2018). These late-decreasing miRNAs played essential roles in muscle hypertrophy. Key DEmiRNAs were also screened in profile 11, which was declining from F45. Among them, the targeted gene *ANGPT4* of chi-miR-767, the targeted gene *POLR3H* of chi-miR-665, and the targeted gene *TEPSIN* of chi-miR-1271-3p were all involved in the enriched functional term “cell differentiation.” Targeted gene *MARK2* of chi-miR-1306-3p regulated muscle stem cell polarity to ensure asymmetric separation (Dumont et al., 2015). Furthermore, five key DEmiRNAs were also selected in the rising spectrum at all stages. The targeted genes of these key DEmiRNAs were involved in the “cytoskeleton organization,” including the targeted genes *GDF7*, *LOC102169433*, and *VSIG10* of chi-miR-150; *ANKRD13D* and *MLPH* of chi-miR-193b-5p; *KCNMA1* and *LRRC73* of chi-miR-361-3p; and *C11H9orf16* of chi-miR-193a and chi-miR-193b-3p. They were also closely related to skeletal muscle development. For example, *FGFR1*, which was targeted by chi-miR-150, was important for FGF-mediated proliferation of skeletal muscle satellite cells and contributed to mitogenic effects (Yablonka-Reuveni et al., 2015). Moreover, *SLIT1*, also targeted by chi-miR-150, drove directional migration and differentiation of Robo2-expressing pioneer myoblasts (Halperin-Barlev and Kalcheim, 2011). In addition, the DEmiRNAs of the three expression profiles were most abundant in the ribosome, whether rising or falling. These data indicated that miRNAs in skeletal muscle development have different effects on ribosomes at different stages.

## Conclusion

In this study, we identified miRNAs in goat skeletal muscle at five fetal stages and two kid stages by RNA-seq and systematically explored temporal trend expression profiles of skeletal muscle development. A total of 421 known miRNAs and 228 goat novel miRNAs were identified in the data. Moreover, 420 DEmiRNAs (364 known miRNAs and 56 goat novel miRNAs) were found in all comparison groups in the seven stages, and the average abundance of 19 miRNAs exceeded 10,000 RPM. The sustained high expression of these miRNAs indicates that they play important cellular biological functions during skeletal muscle development. In addition, DEmiRNAs were clustered into 12 trends and were mainly enriched in three temporal trend expression profiles (profiles 16, 11, and 19). Functional and pathway analyses indicated that the biological pathways regulated by miRNA expression profiles were directly related to temporal changes in goat skeletal muscle development. This study illustrated the role of miRNAs in different goat skeletal muscle stages, which provided valuable information for a better understanding of mammalian skeletal muscle development.

## Data Availability Statement

The datasets generated for this study can be found in the RNA-seq data was available in the SRA database under the accession number PRJNA553597.

## Ethics Statement

This study was carried out following the principles of the Basel Declaration and recommendations of the Guide for the Care and Use of Laboratory Animals (http://grants1.nih.gov/grants/olaw/references/phspol.htm), the ethics committee of Anhui Agricultural University. Anhui Agricultural University Ethics Committee has approved the agreement under permit No. AHAU-AE2017-07.

## Author Contributions

YHL: conceptualization, funding acquisition, methodology, and supervision. YHL and QZ: data curation and formal analysis. QZ and LZ: investigation. MS and LZ: resources. QZ, MS, and LZ: validation. QZ and MS: visualization. QZ: writing – original draft. YHL, YZ, YaL, FF, YM, and XZ: writing – review and editing. All authors contributed to the article and approved the submitted version.

## Conflict of Interest

The authors declare that the research was conducted in the absence of any commercial or financial relationships that could be construed as a potential conflict of interest.

## Footnotes

1 ftp://ftp.ncbi.nlm.nih.gov/genomes/all/GCF/001/704/415/GCF_001704415.1_ARS1/GCF_001704415.1_ARS1_genomic.fna.gz

2 http://rfam.sanger.ac.uk/search/

3 www.omicshare.com/tools

4 https://software.broadinstitute.org/morpheus

5 https://www.ibm.com/support/pages/spss-statistics-210-available-download

## Supplementary Material

FIGURE S1 All trend clustering of DEmiRNAs. (A) We used the STEM to classify these genes into 12 categories. Color denotes enrichment.

TABLE S1 The summary information of small RNA.

TABLE S2 All identified miRNAs and their expression levels in seven skeletal muscle developmental stages.

TABLE S3 Expression levels of differentially expressed miRNA in seven skeletal muscle developmental stages.

TABLE S4 Uniquely expressed miRNAs in each successive comparison group.

TABLE S5 The KEGG enrichment of continuous stage DEmiRNAs.

TABLE S6 The GO terms enrichment of profile 16.

TABLE S7 The KEGG enrichment of profile 16.

TABLE S8 The GO terms enrichment of profile 11.

TABLE S9 The KEGG enrichment of profile 11.

TABLE S10 The GO terms enrichment of profile 19.

TABLE S11 The KEGG enrichment of profile 19.

## References

[B1] Adlakha Y. K. Saini N. (2014). Brain microRNAs and insights into biological functions and therapeutic potential of brain enriched miRNA-128. *Mol. Cancer* 13:33. 10.1186/1476-4598-13-33 24555688 PMC3936914

[B2] Al Jaam B. Heu K. Pennarubia F. Segelle A. Magnol L. Germot A. (2016). Reduced Notch signalling leads to postnatal skeletal muscle hypertrophy in Pofut1cax/cax mice. *Open Biol.* 6:160211. 10.1098/rsob.160211 27628322 PMC5043585

[B3] Alexander M. S. Casar J. C. Motohashi N. Vieira N. M. Eisenberg I. Marshall J. L. (2014). MicroRNA-486-dependent modulation of DOCK3/PTEN/AKT signaling pathways improves muscular dystrophy-associated symptoms. *J. Clin. Invest.* 124 2651–2667. 10.1172/jci73579 24789910 PMC4038577

[B4] Al-Shanti N. Stewart C. E. (2009). Ca2+/calmodulin-dependent transcriptional pathways: potential mediators of skeletal muscle growth and development. *Biol. Rev. Camb. Philos. Soc.* 84 637–652. 10.1111/j.1469-185X.2009.00090.x 19725819

[B5] Antoniali G. Serra F. Lirussi L. Tanaka M. D’Ambrosio C. Zhang S. (2017). Mammalian APE1 controls miRNA processing and its interactome is linked to cancer RNA metabolism. *Nat. Commun.* 8:797. 10.1038/s41467-017-00842-8 28986522 PMC5630600

[B6] Bartel D. P. (2004). MicroRNAs: genomics, biogenesis, mechanism, and function. *Cell* 116 281–297. 10.1016/s0092-8674(04)00045-5 14744438

[B7] Brack A. S. Murphy-Seiler F. Hanifi J. Deka J. Eyckerman S. Keller C. (2009). BCL9 is an essential component of canonical Wnt signaling that mediates the differentiation of myogenic progenitors during muscle regeneration. *Dev. Biol.* 335 93–105. 10.1016/j.ydbio.2009.08.014 19699733 PMC3259687

[B8] Capitanio D. Fania C. Torretta E. Vigano A. Moriggi M. Bravata V. (2017). TCA cycle rewiring fosters metabolic adaptation to oxygen restriction in skeletal muscle from rodents and humans. *Sci. Rep.* 7:9723. 10.1038/s41598-017-10097-4 28852047 PMC5575144

[B9] Chal J. Pourquie O. (2017). Making muscle: skeletal myogenesis *in vivo* and *in vitro*. *Development* 144 2104–2122. 10.1242/dev.151035 28634270

[B10] Chen X. Guo Y. Jia G. Zhao H. Liu G. Huang Z. (2018). Arginine promotes slow myosin heavy chain expression via Akirin2 and the AMP-activated protein kinase signaling pathway in porcine skeletal muscle satellite cells. *J. Agric. Food Chem.* 66 4734–4740. 10.1021/acs.jafc.8b00775 29685038

[B11] Cheng K. K. Akasaki Y. Lecommandeur E. Lindsay R. T. Murfitt S. Walsh K. (2015). Metabolomic analysis of akt1-mediated muscle hypertrophy in models of diet-induced obesity and age-related fat accumulation. *J. Proteome Res.* 14 342–352. 10.1021/pr500756u 25231380 PMC4286153

[B12] Dumont N. A. Wang Y. X. von Maltzahn J. Pasut A. Bentzinger C. F. Brun C. E. (2015). Dystrophin expression in muscle stem cells regulates their polarity and asymmetric division. *Nat. Med.* 21 1455–1463. 10.1038/nm.3990 26569381 PMC4839960

[B13] Dutchak P. A. Estill-Terpack S. J. Plec A. A. Zhao X. Yang C. Chen J. (2018). Loss of a negative regulator of mTORC1 induces aerobic glycolysis and altered fiber composition in skeletal muscle. *Cell. Rep.* 23 1907–1914. 10.1016/j.celrep.2018.04.058 29768191 PMC6038807

[B14] Endo T. (2015). Molecular mechanisms of skeletal muscle development, regeneration, and osteogenic conversion. *Bone* 80 2–13. 10.1016/j.bone.2015.02.028 26453493

[B15] Fang J. H. Zhang Z. J. Shang L. R. Luo Y. W. Lin Y. F. Yuan Y. (2018). Hepatoma cell-secreted exosomal microRNA-103 increases vascular permeability and promotes metastasis by targeting junction proteins. *Hepatology* 68 1459–1475. 10.1002/hep.29920 29637568

[B16] Friedlander M. R. Mackowiak S. D. Li N. Chen W. Rajewsky N. (2012). miRDeep2 accurately identifies known and hundreds of novel microRNA genes in seven animal clades. *Nucleic Acids Res.* 40 37–52. 10.1093/nar/gkr688 21911355 PMC3245920

[B17] Ganassi M. Badodi S. Ortuste Quiroga H. P. Zammit P. S. Hinits Y. Hughes S. M. (2018). Myogenin promotes myocyte fusion to balance fibre number and size. *Nat. Commun.* 9:4232. 10.1038/s41467-018-06583-6 30315160 PMC6185967

[B18] Gorbea C. Mosbruger T. Cazalla D. (2017). A viral Sm-class RNA base-pairs with mRNAs and recruits microRNAs to inhibit apoptosis. *Nature* 550 275–279. 10.1038/nature24034 28976967 PMC5864290

[B19] Guo J. Zhao W. Zhan S. Li L. Zhong T. Wang L. (2016). Identification and expression profiling of miRNAome in goat longissimus dorsi muscle from prenatal stages to a neonatal stage. *PLoS One* 11:e0165764. 10.1371/journal.pone.0165764 27798673 PMC5087842

[B20] Halperin-Barlev O. Kalcheim C. (2011). Sclerotome-derived Slit1 drives directional migration and differentiation of Robo2-expressing pioneer myoblasts. *Development* 138 2935–2945. 10.1242/dev.065714 21653616

[B21] He K. Hu J. Yu H. Wang L. Tang F. Gu J. (2017). Serine/threonine kinase 40 (Stk40) functions as a novel regulator of skeletal muscle differentiation. *J. Biol. Chem.* 292 351–360. 10.1074/jbc.M116.719849 27899448 PMC5217693

[B22] Helwak A. Kudla G. Dudnakova T. Tollervey D. (2013). Mapping the human miRNA interactome by CLASH reveals frequent noncanonical binding. *Cell* 153 654–665. 10.1016/j.cell.2013.03.043 23622248 PMC3650559

[B23] Horak M. Novak J. Bienertova-Vasku J. (2016). Muscle-specific microRNAs in skeletal muscle development. *Dev. Biol.* 410 1–13. 10.1016/j.ydbio.2015.12.013 26708096

[B24] Hornberger T. A. Chu W. K. Mak Y. W. Hsiung J. W. Huang S. A. Chien S. (2006). The role of phospholipase D and phosphatidic acid in the mechanical activation of mTOR signaling in skeletal muscle. *Proc. Natl. Acad. Sci. U.S.A.* 103 4741–4746. 10.1073/pnas.0600678103 16537399 PMC1450240

[B25] Jung H. J. Lee K. P. Kwon K. S. Suh Y. (2019). MicroRNAs in skeletal muscle aging: current issues and perspectives. *J. Gerontol. A Biol. Sci. Med. Sci.* 74 1008–1014. 10.1093/gerona/gly207 30215687 PMC6580686

[B26] Langmead B. Trapnell C. Pop M. Salzberg S. L. (2009). Ultrafast and memory-efficient alignment of short DNA sequences to the human genome. *Genome Biol.* 10:R25. 10.1186/gb-2009-10-3-r25 19261174 PMC2690996

[B27] Lebrasseur N. K. Cote G. M. Miller T. A. Fielding R. A. Sawyer D. B. (2003). Regulation of neuregulin/ErbB signaling by contractile activity in skeletal muscle. *Am. J. Physiol. Cell. Physiol.* 284 C1149–C1155. 10.1152/ajpcell.00487.2002 12519750

[B28] Li L. Fan C. M. (2017). A CREB-MPP7-AMOT regulatory axis controls muscle stem cell expansion and self-renewal competence. *Cell Rep.* 21 1253–1266. 10.1016/j.celrep.2017.10.031 29091764 PMC5710848

[B29] Ling Y. H. Sui M. H. Zheng Q. Wang K. Y. Wu H. Li W. Y. (2018). miR-27b regulates myogenic proliferation and differentiation by targeting Pax3 in goat. *Sci. Rep.* 8:3909. 10.1038/s41598-018-22262-4 29500394 PMC5834623

[B30] Liu Z. Li C. Li X. Yao Y. Ni W. Zhang X. (2019). Expression profiles of microRNAs in skeletal muscle of sheep by deep sequencing. *Asian Austral. J. Anim. Sci.* 32 757–766. 10.5713/ajas.18.0473 30477295 PMC6498074

[B31] Ma G. Wang Y. Li Y. Cui L. Zhao Y. Zhao B. (2015). MiR-206, a key modulator of skeletal muscle development and disease. *Int. J. Biol. Sci.* 11 345–352. 10.7150/ijbs.10921 25678853 PMC4323374

[B32] Mao X. Cai T. Olyarchuk J. G. Wei L. (2005). Automated genome annotation and pathway identification using the KEGG Orthology (KO) as a controlled vocabulary. *Bioinformatics* 21 3787–3793. 10.1093/bioinformatics/bti430 15817693

[B33] Meister G. (2007). miRNAs get an early start on translational silencing. *Cell* 131 25–28. 10.1016/j.cell.2007.09.021 17923084

[B34] Moriya N. Miyazaki M. (2018). Akt1 deficiency diminishes skeletal muscle hypertrophy by reducing satellite cell proliferation. *Am. J. Physiol. Regul. Integr. Comp. Physiol.* 314 R741–R751. 10.1152/ajpregu.00336.2017 29443546

[B35] Murach K. A. Fry C. S. Kirby T. J. Jackson J. R. Lee J. D. White S. H. (2018). Starring or supporting role? Satellite cells and skeletal muscle fiber size regulation. *Physiology* 33 26–38. 10.1152/physiol.00019.2017 29212890 PMC5866409

[B36] Ost M. Keipert S. van Schothorst E. M. Donner V. van der Stelt I. Kipp A. P. (2015). Muscle mitohormesis promotes cellular survival via serine/glycine pathway flux. *FASEB J.* 29 1314–1328. 10.1096/fj.14-261503 25491309

[B37] Roy S. Bantel H. Wandrer F. Schneider A. T. Gautheron J. Vucur M. (2017). miR-1224 inhibits cell proliferation in acute liver failure by targeting the antiapoptotic gene Nfib. *J. Hepatol.* 67 966–978. 10.1016/j.jhep.2017.06.007 28645739

[B38] Shang F. F. Zhao W. Zhao Q. Liu J. Li D. W. Zhang H. (2013). Upregulation of eIF-5A1 in the paralyzed muscle after spinal cord transection associates with spontaneous hindlimb locomotor recovery in rats by upregulation of the ErbB, MAPK and neurotrophin signal pathways. *J. Proteomics* 91 188–199. 10.1016/j.jprot.2012.12.002 23238062

[B39] Song C. Yang J. Jiang R. Yang Z. Li H. Huang Y. (2019). miR-148a-3p regulates proliferation and apoptosis of bovine muscle cells by targeting KLF6. *J. Cell Physiol.* [Epub ahead of print]. 10.1002/jcp.28232 30793769

[B40] Tan T. Scholz P. M. Weiss H. R. (2010). Hypoxia inducible factor-1 improves the negative functional effects of natriuretic peptide and nitric oxide signaling in hypertrophic cardiac myocytes. *Life Sci.* 87 9–16. 10.1016/j.lfs.2010.05.002 20470788 PMC2900401

[B41] Tidyman W. E. Lee H. S. Rauen K. A. (2011). Skeletal muscle pathology in Costello and cardio-facio-cutaneous syndromes: developmental consequences of germline Ras/MAPK activation on myogenesis. *Am. J. Med. Genet. C Semin. Med. Genet.* 157c 104–114. 10.1002/ajmg.c.30298 21495178

[B42] Title A. C. Hong S. J. Pires N. D. Hasenohrl L. Godbersen S. Stokar-Regenscheit N. (2018). Genetic dissection of the miR-200-Zeb1 axis reveals its importance in tumor differentiation and invasion. *Nat. Commun.* 9:4671. 10.1038/s41467-018-07130-z 30405106 PMC6220299

[B43] Wang H. Noulet F. Edom-Vovard F. Tozer S. Le Grand F. Duprez D. (2010). Bmp signaling at the tips of skeletal muscles regulates the number of fetal muscle progenitors and satellite cells during development. *Dev. Cell.* 18 643–654. 10.1016/j.devcel.2010.02.008 20412778

[B44] Wang J. Tan J. Qi Q. Yang L. Wang Y. Zhang C. (2018). miR-487b-3p suppresses the proliferation and differentiation of myoblasts by targeting IRS1 in skeletal muscle myogenesis. *Int. J. Biol. Sci.* 14 760–774. 10.7150/ijbs.25052 29910686 PMC6001677

[B45] Wang Y. Zhang C. Fang X. Zhao Y. Chen X. Sun J. (2014). Identification and profiling of microRNAs and their target genes from developing caprine skeletal Muscle. *PLoS One* 9:e96857. 10.1371/journal.pone.0096857 24818606 PMC4018397

[B46] Wen M. Shen Y. Shi S. Tang T. (2012). miREvo: an integrative microRNA evolutionary analysis platform for next-generation sequencing experiments. *BMC Bioinformatics* 13:140. 10.1186/1471-2105-13-140 22720726 PMC3410788

[B47] Wust S. Drose S. Heidler J. Wittig I. Klockner I. Franko A. (2018). Metabolic maturation during muscle stem cell differentiation is achieved by miR-1/133a-mediated inhibition of the Dlk1-Dio3 mega gene cluster. *Cell Metab.* 27 1026.e6–1039.e6. 10.1016/j.cmet.2018.02.022 29606596

[B48] Yablonka-Reuveni Z. Danoviz M. E. Phelps M. Stuelsatz P. (2015). Myogenic-specific ablation of Fgfr1 impairs FGF2-mediated proliferation of satellite cells at the myofiber niche but does not abolish the capacity for muscle regeneration. *Front. Aging Neurosci.* 7:85. 10.3389/fnagi.2015.00085 26074812 PMC4446549

[B49] Young M. D. Wakefield M. J. Smyth G. K. Oshlack A. (2010). Gene ontology analysis for RNA-seq: accounting for selection bias. *Genome Biol.* 11:R14. 10.1186/gb-2010-11-2-r14 20132535 PMC2872874

[B50] Zhao Q. Kang Y. Wang H. Y. Guan W. J. Li X. C. Jiang L. (2016). Expression profiling and functional characterization of miR-192 throughout sheep skeletal muscle development. *Sci. Rep.* 6:30281. 10.1038/srep30281 27452271 PMC4958965

[B51] Zhao Y. J. Chang Q. Jiang H. Z. (2012). A preliminary study on the fetal development of Liaoning cashmere goats. *China Herbivore Sci.* 32 20–23.

[B52] Zhou L. Chen J. Li Z. Li X. Hu X. Huang Y. (2010). Integrated profiling of microRNAs and mRNAs: microRNAs located on Xq27.3 associate with clear cell renal cell carcinoma. *PLoS One* 5:e15224. 10.1371/journal.pone.0015224 21253009 PMC3013074

[B53] Zhu M. J. Ford S. P. Nathanielsz P. W. Du M. (2004). Effect of maternal nutrient restriction in sheep on the development of fetal skeletal muscle. *Biol. Reprod.* 71 1968–1973. 10.1095/biolreprod.104.034561 15317692

