# Supplementary figures and images for: RNA-Seq Reveals miRNA Role Shifts in Seven Stages of Skeletal Muscles in Goat Fetuses and Kids
Source: Front Genet. 2020 Jul 7;11:684. doi: 10.3389/fgene.2020.00684 (PMC7358459; doi:10.3389/fgene.2020.00684)

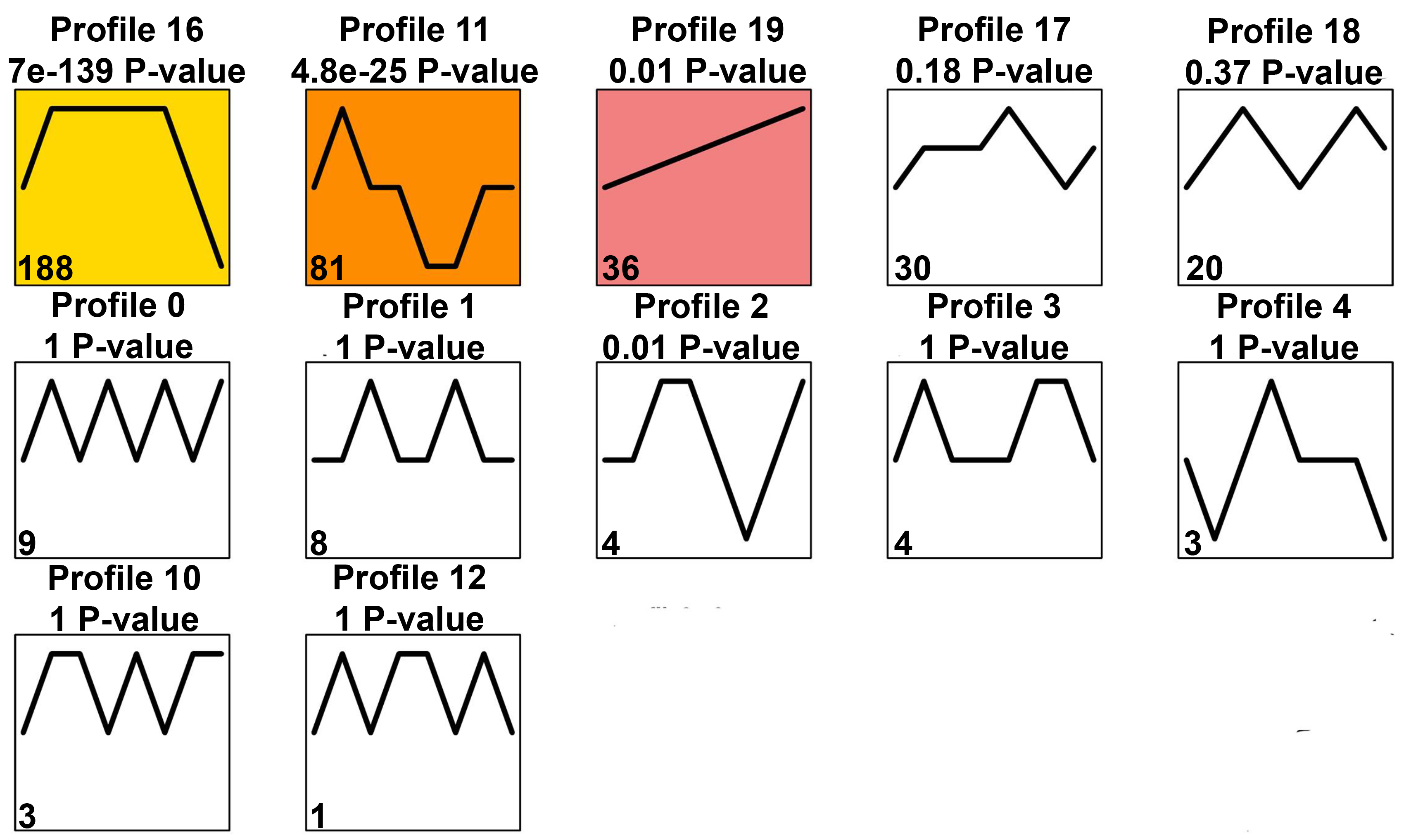

Supplement: FIGURE S1 — All trend clustering of DEmiRNAs. (A) We used the STEM to classify these genes into 12 categories. Color denotes enrichment. [file Image_1.JPEG]
